# Odor identity influences tracking of temporally patterned plumes in Drosophila

**DOI:** 10.1186/1471-2202-12-62

**Published:** 2011-06-27

**Authors:** Parthasarathy Krishnan, Brian J Duistermars, Mark A Frye

**Affiliations:** 1 UCLA Department of Integrative Biology and Physiology, 621 Charles E. Young Dr. South, Box 951606, Los Angeles, CA 90095-1606, USA; 2 Howard Hughes Medical Institute, 621 Charles E. Young Dr. South, Box 951606, Los Angeles, CA 90095-1606, USA

## Abstract

**Background:**

Turbulent fluid landscapes impose temporal patterning upon chemical signals, and the dynamical neuronal responses to patterned input vary across the olfactory receptor repertoire in flies, moths, and locusts. Sensory transformations exhibit low pass filtering that ultimately results in perceptual fusion of temporally transient sensory signals. For example, humans perceive a sufficiently fast flickering light as continuous, but the frequency threshold at which this fusion occurs varies with wavelength. Although the summed frequency sensitivity of the fly antenna has been examined to a considerable extent, it is unknown how intermittent odor signals are integrated to influence plume tracking behavior independent of wind cues, and whether temporal fusion for behavioral tracking might vary according to the odor encountered.

**Results:**

Here we have adopted a virtual reality flight simulator to study the dynamics of plume tracking under different experimental conditions. Flies tethered in a magnetic field actively track continuous (non-intermittent) plumes of vinegar, banana, or ethyl butyrate with equal precision. However, pulsing these plumes at varying frequency reveals that the threshold rate, above which flies track the plume as if it were continuous, is unique for each odorant tested. Thus, the capability of a fly to navigate an intermittent plume depends on the particular odorant being tracked during flight. Finally, we measured antennal field potential responses to an intermittent plume, found that receptor dynamics track the temporal pattern of the odor stimulus and therefore do not limit the observed behavioral temporal fusion limits.

**Conclusions:**

This study explores the flies' ability to track odor plumes that are temporally intermittent. We were surprised to find that the perceptual critical fusion limit, determined behaviorally, is strongly dependent on odor identity. Antennal field potential recordings indicate that peripheral processing of temporal cues faithfully follow rapid odor transients above the rates that can be resolved behaviorally. These results indicate that (1) higher order circuits create a perceptually continuous signal from an intermittent sensory one, and that (2) this transformation varies with odorant rather than being constrained by sensory-motor integration, thus (3) offering an entry point for examining the mechanisms of rapid olfactory decision making in an ecological context.

## Background

*Drosophila melanogaster *is a cosmopolitan generalist that seeks the sources of diverse odorants [1,2]. Tracking odors in flight is supported in part by bilateral spatial comparisons signal intensity across the antennae [3]. However, due to fluid turbulence, the pattern of sensory exposure to odors in natural olfactory plumes also contains a high degree of temporal fluctuation [4,5]. Olfactory receptor neurons (ORNs) and second order projection neurons (PNs) show temporal patterning which varies across receptors for a given odorant and across odors for a given receptor [6-8]. The identity of odors influences behavioral responses. During flight in *Drosophila*, behavioral responses to odors depend upon which specific ORNs are activated [9]. The variation in temporal coding by ORNs coupled with the complex mapping of ORN input onto behavioral responses, would seem to pose a challenge to the tracking algorithm, yet might be mitigated in part by temporal fusion to enhance perceptual salience.

Neuronal mechanisms of peripheral olfactory coding are well studied in *D. melanogaster *[10]. The odorant receptor (OR) repertoire selectively responds to a host of volatile chemical cues, which can be broadly classified as emerging from food, predators or a potential mating partner. Recent evidence suggests that these odorant receptors are comprised of heterodimeric cation channels, which could support the encoding of rapid odor transients [11]. Electrophysiological studies in *Drosophila *have shown that ORs encode the temporal dynamics of an odor stimulus peaking between 1-10 Hz and falling off by approximately 100 Hz, thus defining an upper limit for the rate of information processing by the fly olfactory system [12]. In locusts and moths, neuronal response dynamics mimic temporal aspects of the stimulus pattern [7,13]. PNs in moth macroglomular complex follow stimulus pulses up to about 10 Hz [14]. However, relative to spatial coding of the olfactory repertoire, virtually nothing is known about how the temporal dynamics of an olfactory signal are integrated into fly behavior.

What is the rate at which a temporally intermittent olfactory stimulus is perceived as continuous, and is therefore behaviorally tracked as if it were a steady stream? Temporal dynamics of sensory systems can be characterized by the Critical Flicker Fusion Frequency (CFFF), or the rate at which multiple discrete pulses of a stimulus are perceived as a continuous signal [15]. In the case of lobster chemoreception the CFFF is 5 Hz, corresponding to a stimulus integration time of 200 ms [15]. In the human visual system, the flicker-fusion frequency is wavelength-dependent, with longer wavelengths having lower CFFF [16].

We have sought to investigate how active tracking of a patterned odor plume varies with stimulus frequency and odor type. We used a virtual-reality plume simulator in which a magnetically tethered hungry fruit fly is free to rotate in the yaw plane of an arena capable of delivering precise visual and olfactory stimuli. By virtue of its design, the odor source in the arena is contained within a narrow low-velocity plume that does not elicit sensory adaptation, as there is no persistent odor field of constant concentration. In order to track the plume effectively, the fly must maintain its mean position within a narrow angular region of the arena, but makes small shifts in heading, back and forth. Although flies track a continuous plume of vinegar, banana, or ethyl butyrate equally well, pulsing these odors into intermittent plumes causes changes in tracking behavior that are unique for each odor. Thus, we have determined a CFFF threshold value for each odorant tested and found it to vary with odor identity in a manner qualitatively similar to the wavelength dependence of human visual flicker-fusion threshold.

To begin to examine the mechanism of the fly's CFFF, we made field potential recordings from the antennal funiculus in response to a temporally structured plume. Incidentally, this also served as a control for our stimulus delivery apparatus. We found that the relatively low behavioral flicker-fusion threshold values cannot be explained by the rather rapid olfactory transients that are encoded by the sensory system.

## Results

### In the magnetically tethered flight assay spatio-temporal properties of the odor plume are experimentally controlled

**Figure 1:**
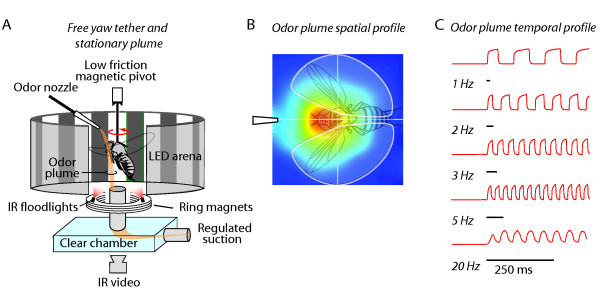
**Magnetically tethered flight assay and spatio-temporal measurement of odor plume tracking**. (A) A hungry fly is tethered within a magnetic field that allows her to steer freely in the yaw plane. A wraparound LED display arena displays either a high contrast stationary grating pattern or moving features to "drag" the animal toward the odor nozzle. The nozzle delivers a smooth narrow odor plume which is drawn downward by an acrylic suction chamber. The fly's angular heading in the arena is tracked with IR video and software written in Matlab. (B) A miniature-photoionization detector (mini-PID) was used to make odor intensity measurements within a 9 mm^2 ^space occupied by the fly. Odor intensity, in arbitrary units, is plotted in pseudo-color. Each point in the grid was sampled 11 different times below the plane of a fly in flight and the resulting data was smoothed using piecewise linear interpolation in Matlab. (C)The plume was pulsed with a solenoid valve system, and the temporal fidelity of the pulse train was verified with mini-PID measurements made at the location of the tethered fly.

The aim of this study was to uncover how variation in the temporal dynamics of odor stimuli (and therefore receptor activation) influences the temporal integration required for active behavioral tracking. We assumed that in order to track a food odor plume, the fly must integrate or fuse temporal modulations in the signal, and that if we imposed sufficiently large intervals between odor pulses, the tracking behavior would degrade. To test this prediction, we tethered a hungry fly within a flight arena in which a magnetic field enables free movement in the yaw plane and changes in its heading are tracked with video (Figure 1A). Enveloping the fly's center of rotation, a cylindrical LED panel array displays visual patterns. Oscillating a narrow vertical bar horizontally back and forth across one position in the arena induces a robust visual fixation reflex thereby 'dragging' the fly into a specified position. At the visual setpoint is an odor nozzle, which delivers a mass flow regulated stream of vapor through a solenoid valve to switch between odor and water vapor. The plume is drawn vertically downward by continuous suction from beneath (Figure 1A). We use the bar fixation reflex to position the fly in front of the odor nozzle, directly in the plume, at the start of each experimental trial.

To determine the spatial and temporal properties of the odor plume we used a miniature photoionization detector (PID) capable of measuring volatile gas concentrations over a wide range of intensities with millisecond resolution. PIDs are used extensively in olfaction research to measure the fine scale temporal and spatial distribution of odor signals [8,12,17]. We sampled a spatial grid spanning the odor port in our behavioral setup to show that the area enveloping the head of the fly in flight receives the highest intensity of odor molecules (Figure 1B). We also used the PID to corroborate the fidelity of the solenoid switching system and confirmed that our apparatus is capable of delivering odor pulses at 1 Hz to 20 Hz that return to baseline intensity between each switching cycle (Figure 1C).

### Flies track continuous plumes of three diverse test odors equally well

**Figure 2:**
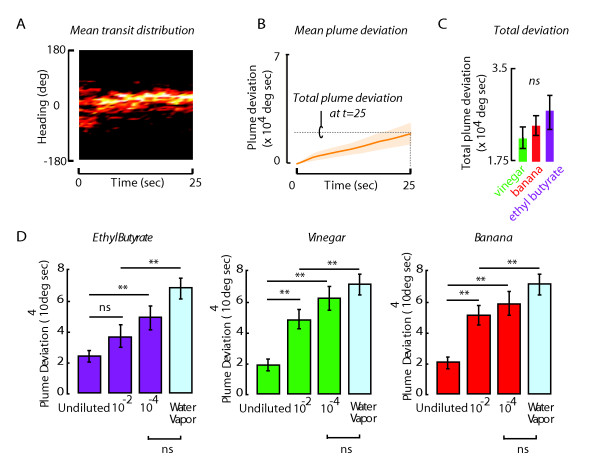
**Flies track constant odor plumes of different odors equally well**. (A) Mean distribution of the flight trajectories of 25 flies presented with a continuous plume of apple cider vinegar. The frequency of occurrence is spatially coded with pseudo-color. The tight distribution centered at zero degrees indicates the precision and accuracy of plume tracking during flight. (B) The same trajectories as (A) are analyzed for cumulative plume deviation, the angular magnitude of deviation from the plume cumulatively summed over the trial. (C) Total plume deviation is similar across three different odors: ethyl butyrate, apple cider vinegar and banana, indicating equal tracking accuracy (t-test p > 0.05; n > = 25 flies for each odor). Error bars indicate SEM. (D) Total plume deviation was assessed after challenging flies to track different odor intensities of ethyl butyrate, vinegar and banana. The results indicate that tracking performance does not improve by decreasing odor concentration, but only degrades, and is not significantly different from water vapor control at odor concentrations diluted by two orders of magnitude (10^-4^) for all three odors tested. t-test was used to test for significance, ** denotes p < 0.01 and ns denotes p > 0.05; n > = 10 flies for each odor). Error bars indicate SEM.

In a continuous plume of attractive apple cider vinegar, flies actively track the odor gradient, directing their mean flight heading towards the odor nozzle (Figure 2A). We quantify tracking performance by measuring the cumulative difference between the fly's angular heading and the odor nozzle, centered at zero degrees by our convention. Positive values indicate a turn to the right, or clockwise from the nozzle and *vice versa *for negative angle values. Specifically, we used the *cumsum *function in Matlab to compute the cumulative summation of angles between the fly's heading and the odor nozzle. This metric differs from a static mean value in that it increases for both mean offset relative to the nozzle and steering back and forth across the nozzle, with zero change in mean heading. Higher values indicate greater displacement from the nozzle, or poorer overall tracking. This value cannot remain constant unless the flies are centered exactly in front of the nozzle and makes no steering movements. However, flies do not tend to stay centered in one place passively but instead make fine-scale adjustments in a back-and-forth manner within the plume resulting in a progressively increasing plume deviation. A scalar measure of performance for a single trial is indicated by the final plume deviation value in the trial, indicating the total angular displacement from the nozzle over the course of the trial (Figure 2B).

**Figure 3:**
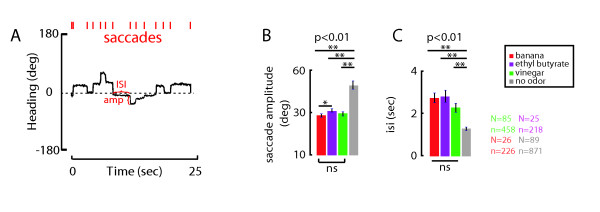
**Saccade dynamics are invariant across odors**. (A) Sample individual flight trajectory visually illustrating the parameters of saccade amplitude and inter-saccade interval (ISI). Raster plot shows rate of saccade events over time. (B) Saccade amplitude and ISI (C) are different from water control across all odors tested. Error bars indicate SEM and the sample size for each odor is color matched.

Flies track headspace vapor of all three odorants with statistically similar plume deviation (Figure 2C). This result indicates that flies maintain similarly stable heading with respect to the odor nozzle for all three test odorants delivered from the nozzle continuously. When exploring the arena or to maintain their heading at the nozzle, flies make rapid reorientations in heading called body saccades (Figure 3A). We measured mean saccade amplitude and inter-saccade interval (ISI), and found that for the three odors, saccade amplitude was reduced, and ISI increased significantly from the water vapor control (Figures 3B,C). This is consistent with the finding that an odor plume stabilizes flight heading in part through reducing saccade amplitude and increasing their time interval [18]. None of these three odors, delivered in a continuous plume produced behavioral responses that were qualitatively or quantitatively unique - all three odors seem to elicit similar smooth tracking and modulation of saccade amplitude and timing.

To facilitate robust tracking behavior, we delivered plumes at the highest gaseous concentration possible, from the headspace of undiluted solution. In odor field assays, particularly for apple cider vinegar, undiluted headspace vapor is highly aversive [19], yet in our apparatus, these headspace concentrations are always highly attractive. The apparent paradox is explained by the design of our olfactometer, which delivers a plume with a very low flow rate to isolate olfactory cues from mechanosensory upwind tracking reflexes common to insects such flies and moths [1,20]. More importantly, the low flow rate also produces a spatially narrow plume that never envelopes both antennae with a constant high intensity signal, as experienced in odor field assays. Because the flow rate is low and the plume is narrow, we reason that the flies are therefore experiencing physiologically realistic odor concentrations, and if they weren't then we would expect to see some compromise to tracking or active repulsion as concentration is increased. Yet we have never observed improved tracking performance after reducing odor concentration maximum, but rather only degraded tracking which becomes not significantly different from water vapor control at a stimulus concentration diluted by two orders of magnitude (10^-4^) for all three odors tested (Figure 2D). The attractive feature of our design is that it elicits highly robust spatial tracking behavior - flies target the plume center with remarkable persistence and precision. The requisite compromise is that we do not produce odor field conditions and thus are unable to present typically attractive odors at concentrations sufficient to elicit repulsion.

### Behavioral flicker-fusion is determined by odor identity

Our results from the continuous plume show that each of the three test odorants (Figures 2, 3) influences the control of stable flight heading similarly. Having satisfied this crucial prerequisite, we next assessed the influence of temporally pulsing the different test odors. We first estimated the relevant test frequency range for each odorant by presenting a sweep of decreasing pulse frequency over the 25-second trial (additional file 1). We reasoned that as the plume became progressively more fragmented, the temporal integration of odor fluctuations would fail and tracking error would rise. We did this experiment only to coarsely estimate which frequency values to test in trials of fixed frequency.

**Figure 4:**
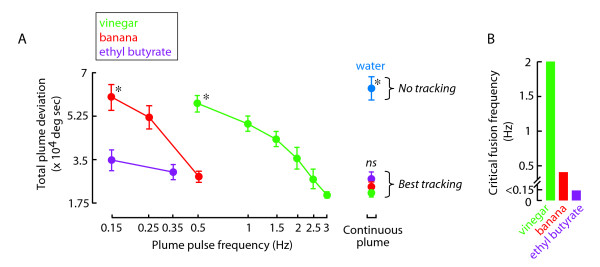
**Determination of the Critical Flicker Fusion Frequency (CFFF) for each odorant**. (A) Flies received vinegar vapor pulsed at 0.5 to 3 Hz at intervals of 0.5 Hz; for banana we tested 0.15, 0.25 and 0.5 Hz; for ethyl butyrate vapor we tested at 0.15 and 0.35 Hz. The test order was randomized and included a water vapor control. Points are means, error bars indicate SEM. (B) Critical flicker fusion frequency was defined as the highest stimulus frequency for which mean plume deviation statistically differed from the continuous plume. The critical fusion frequency was 2 Hz for vinegar, 0.5 Hz for banana, and <0.15 Hz for ethyl butyrate (t-test p < 0.05).

The sweep experiment provided the following frequency values to test individually: vinegar,0.5 to 3 Hz at intervals of 0.5 Hz; banana, 0.15, 0.25 and 0.5 Hz; and since ethyl butyrate responses seemed consistent over a wide range of frequencies, we chose to measure only two frequencies -0.15 and 0.35 Hz. Each pulse frequency was then presented in random order for 25-second experimental trials. By measuring total plume deviation at the end of each trial, we plotted psychometric curves for tracking error as a function of plume pulse frequency (Figure 4A). For both vinegar and banana odor, the curves span the entire range of tracking performance, from strong tracking that was statistically identical to the continuous odor stream to essentially no tracking that was statistically identical to the water vapor control (Figure 4A). Vinegar elicits tracking performance that is not significantly different from the continuous plume for pulse rates above 2.5 Hz, but degrades monotonically, and at 0.5 Hz the animals do not track the plume any better than the water control (Figure 4A). By contrast, flies track a banana plume pulsed at 0.5 Hz as if it was continuous, and their tracking does not degrade to the no-odor water condition until the pulse rate falls to 0.15 Hz (Figure 4A). By contrast, for ethyl butyrate, a low very pulse rate of 0.35 Hz elicits tracking as robust as if the plume were delivered in a continuous stream. The results show that the pulse frequencies that can be tracked effectively are distinct for the three odors (Figure 4A).

From these data, we estimated the critical fusion frequency for each odorant, which represents the first stimulus frequency at which the mean deviation in heading differed significantly from the continuous plume. In other words, the critical fusion frequency for odor represents the first tested threshold pulse rate above which flies track the plume in a manner indistinguishable from the continuous stimulus. The critical fusion frequency for vinegar was 2 Hz, for banana was 0.5 Hz, and for ethyl butyrate was less than 0.15 Hz (Figure 4B). These results indicate that a flies' ability to integrate temporal intensity fluctuations within a plume varies with odor identity. Variation in the perceived intensity or vapor pressure of the different stimuli cannot explain these findings because the three odorants are tracked equally well when presented in a continuous stream.

### Odor-off responses to ethyl butyrate reveal distinct temporal filtering

We were intrigued by the extremely low-pass temporal characteristics of the ethyl butyrate tracking response (Figure 4A). Therefore, we next measured the influence of abruptly truncating the odor plume after three seconds of continuous exposure. We reasoned that an impulsive odor-off stimulus would reveal the dynamical behavioral latency to losing plume contact entirely.

**Figure 5:**
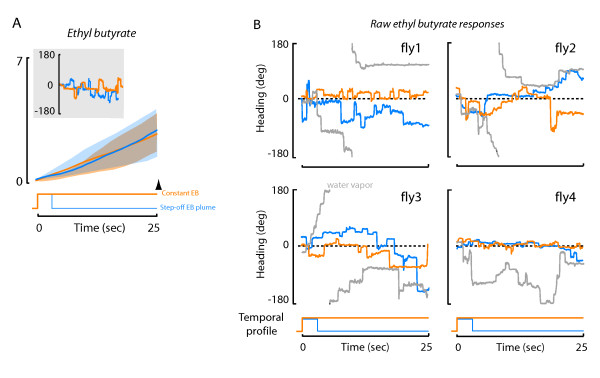
**Responses to abrupt termination of the ethyl butyrate plume**. Comparison of mean plume deviation for flies that received either a continuous plume of ethyl butyrate(orange) or an odor plume that was switched to water vapor after 3 seconds (blue). One standard deviation above and below the mean is indicated with the shaded envelope. (A) Flies that received ethyl butyrate had a mean plume deviation that was not significantly different from flies that received the continuous plume (t-test p > 0.05). n > = 25 flies for each treatment. Inset show example flight trajectories from an individual fly that received both treatments. (B) Exemplar flight trajectories from four individual flies that each received (i) a continuous plume of ethyl butyrate (orange), (ii) a truncated ethyl butyrate plume (switched to water vapor after 3 seconds, blue), and (iii) continuous water vapor control (grey).

Remarkably, over the 25 second time course of our experiments, tracking behavior persisted as if the plume were still active. The trajectory of plume deviation was statistically indistinguishable between the truncated and continuous plumes (Figure 5A). This peculiar result suggests two things: (1) that the neural representation of ethyl butyrate far outlasts the stimulus duration, and (2) that flies can somehow actively orient toward the nozzle in the total absence of a spatial odor gradient. To highlight this interesting phenomenon, we plotted raw data from four individual flies, each exposed to three treatments: a continuous plume of water vapor, a continuous plume of ethyl butyrate, and a truncated plume of ethyl butyrate. In no case did individual flies actively track the water vapor plume, but rather meandered around the arena as if there were no local stimulus at all (Figure 5B). Each fly tracked both the continuous and truncated ethyl butyrate plumes with similar accuracy and precision (Figure 5B). The raw individual trials reveal that within individuals, the fine structure of heading trajectory is nearly identical for the continuous and truncated ethyl butyrate plumes (Figure 5B). When flies were subjected to a similar truncated plume of either vinegar or banana, we found that, as expected, the flies steered away from the odor nozzle within a few seconds of abrupt odor loss (data not shown). In free flight experiments, the latency to the end of tracking behavior observed after a banana odor plume loss in *D.melanogaster *is an average 290 ms[1]. It is reasonable that in free flight an odor-off latency would be much shorter than in our experiments because in free flight the animals are encountering many other sensory signals that vary in time, such as visual cues from optic flow. By contrast, we are showing a longer odor-off latency because none of the other sensory conditions change in a manner that might elicit steering maneuvers. In moths, there is a considerably wider range in latencies observed in response to a loss of a pheromone plume, 150-220 ms in *Grapholitha molesta*, 490 ms in *Manducasexta *to about 1 second in *Lymantria dispar*[21,22]. The results that we observe with ethyl butyrate are unique in that the behavioral latency to odor-off outlasts the duration of our experiments.

### Behavioral response to ethyl butyrate is not an artifact of the odor delivery system

**Figure 6:**
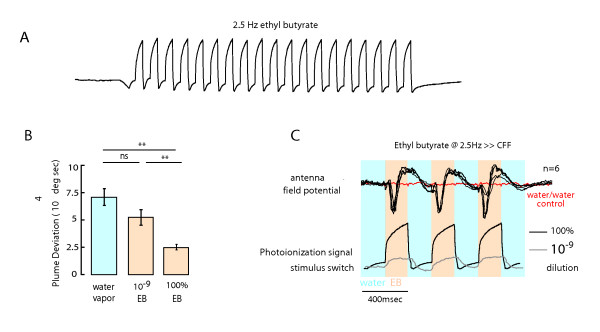
**Odor delivery system faithfully reproduces the temporal stimuli**. (A) A miniature photoionization detector was used to measure the temporal fidelity of a 2.5 Hz train of ethyl butyrate. Transients recover to baseline levels during, suggesting that the olfactometer faithfully produced temporally precise stimuli. (B) Flies were challenged to track a 10^-9 ^dilution of ethyl butyrate (a stimulus that registered a distinct signal recorded by PID measurements as shown in (C). At this dilution, the plume deviation was not significantly different than the response to water vapor alone. However, the responses to flies tracking undiluted ethyl butyrate were significantly different than flies receiving either a 10^-9 ^dilution of ethyl butyrate or water vapor. (t-test p < 0.01). n = 9 flies. (C) Distinct electrophysiological responses were observed to each pulse of ethyl butyrate which fully recovered to baseline when the solenoid valve controlling the water vapor was switched on. This refutes the possibility of any odor-induced contamination between ethyl butyrate pulses that might be below the PID detection limit. Shown are overlaid AFP traces recorded from 6 different preparations.

The peculiar persistent tracking behavior was a concern to us because ethyl butyrate, an ester, could easily become adsorbed to the delivery nozzle such that the discrete pulse train was contaminated by this artifact and thereby 'dribble' odor stimuli between pulses. We tested for this possible contamination in several ways. First, rather than use a tracer gas, we took mini-PID measurements of the ethyl butyrate plume directly. The ionization potential of ethyl butyrate is higher by comparison to ethanol (our standard tracer gas, Figure 1C), and therefore the signal to noise ratio is lower. Nevertheless, the results show that ethyl butyrate transients recover to baseline levels for each discrete epoch during a 2.5 Hz pulse train (Figure 6A), and this frequency far exceeds the temporal resolution of the behavior (Figures 4A,B). Second, we performed a serial dilution experiment to show that at 10^-9 ^dilution of ethyl butyrate, flies no longer track a continuous plume more effectively than they do a water control (Figure 6B). At this concentration, however, the PID shows full recovery to baseline during a pulse train (Figure 6C) thus indicating that the instrument is more sensitive than the fly's tracking behavior. However, the fly's olfactory receptors have been shown to respond at extremely low odorant concentrations [6], and therefore we also performed field potential recordings or electroantenograms (EAGs) from the antennal funiculus (third antennal segment of the antenna) in response to ethyl butyrate pulses in our behavioral apparatus. These recordings reflect the summed responses of a population of ORNs and are used as a conservative estimate of OR activity [13,23]. We pulsed the signal at 2.5 Hz, which is far higher than the behavioral resolution indicated by the tracking results (Figure 4), yet the field potential responses fully recover to baseline between pulses (Figure 6C). This shows conclusively that the olfactometer is delivering clean air between odor pulses. Taken together these three complementary experiments confirm that our olfactometer is faithfully delivering ON-OFF odor transients at specified frequencies, and there is no evidence of leakage or contamination between odor pulses. Additionally, the observation that field potential recordings faithfully track temporal dynamics upward of 2.5 Hz, but the behavior does not, allows us to conclude that the persistent representation of this particular odorant must persist downstream of the peripheral sensory circuits.

### Ethyl butyrate responses engage well-known multi-sensory integration circuits

**Figure 7:**
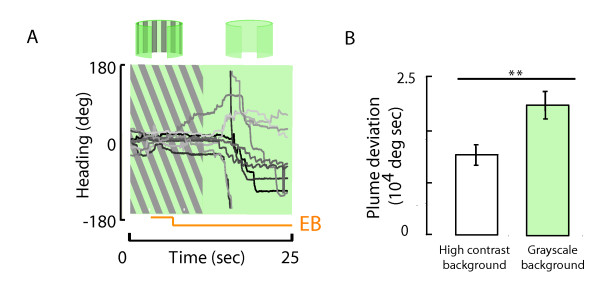
**Flies actively integrate cross-modal visual cues while tracking the terminated ethyl butyrate plume**. (A) After flies were 'visually' dragged near the odor nozzle, the ethyl butyrate plume was turned on and terminated after 3 seconds. The arena pattern was set to high contrast vertical stripes. Flies could no longer track the ethyl butyrate plume upon switching to a grayscale pattern after 12 seconds from the start of the experiment. Shown are 5 exemplar individual flight trajectories. (B) Plume deviation, a metric used to assess quality of plume tracking, was computed when the grayscale pattern was switched on and compared to the extent of plume deviation when the arena displayed high-contrast visual stripes. The two values were found to be significantly different (t-test p < 0.01). n = 20 flies.

Upon termination of the ethyl butyrate plume, flies persist in remaining oriented at the arena location of the odor nozzle, and as such produce a plume deviation trajectory identical to the continuous plume (Figure 5A). This is not a passive stabilization response; rather, individual flies can be seen to make small amplitude saccades, back and forth, throughout the trial even though there is no odor gradient to be tracked by the antennae (Figure 5B). It is possible that the apparent persistence of an odor stimulus engages well-known cross-modal activation of optomotor responses in order to keep a fly actively oriented within a visual set point at the fictive plume. To examine this hypothesis, we positioned flies in front of the odor nozzle in the usual way, and terminated the plume after three seconds, repeating the experiments from Figure 5. However, after three additional seconds, the visual panorama was switched from a high contrast horizontal grating that generates rich motion cues when the animal turns, to an equiluminant featureless grayscale background that generates no motion cues at all when the animal moves. Switching to the uniform featureless panorama causes flies to quickly lose their ability to remain centered toward the inactive odor nozzle, and they begin to veer away (Figure 7A). As a result, flies presented with a high contrast panorama show significantly lower plume deviation than do flies presented with the uniform featureless panorama, even though neither of these groups was encountering any odor signal at all after the first three seconds of the trial (Figure 7B). This result suggests that the ethyl butyrate odor produces a lasting perception that itself engages optomotor stabilization responses as if the plume were still being encountered.

## Discussion

Natural odor plumes are spatially and temporally intermittent [4,17]. In this study, we used a magnetic tether system and a high performance olfactometer that presents an odor plume without mechanosensory wind cues to examine how fruit flies integrate a spatially restricted and temporally patterned odor stimulus for active plume tracking during flight (Figures 1, 2). We tested the hypothesis that tracking performance varies according to both the temporal frequency of exposure and odor type. We systematically varied the temporal pulse rate of each of three odors and, to our surprise, found that the precision with which a fly can track a patterned plume varies more across odor types than across temporal frequencies (Figure 4). One odorant, ethyl butyrate, produced tracking responses that far outlasted the stimulus (Figure 5). Active tracking of the 'phantom plume' cannot be attributed to the performance of the olfactometer (Figure 6), but can be attributed in part to olfactory enhanced optomotor responses that stabilize the fly's heading (Figure 7).

### Behavioral flicker-fusion thresholds are shaped downstream of the antenna

**Figure 8:**
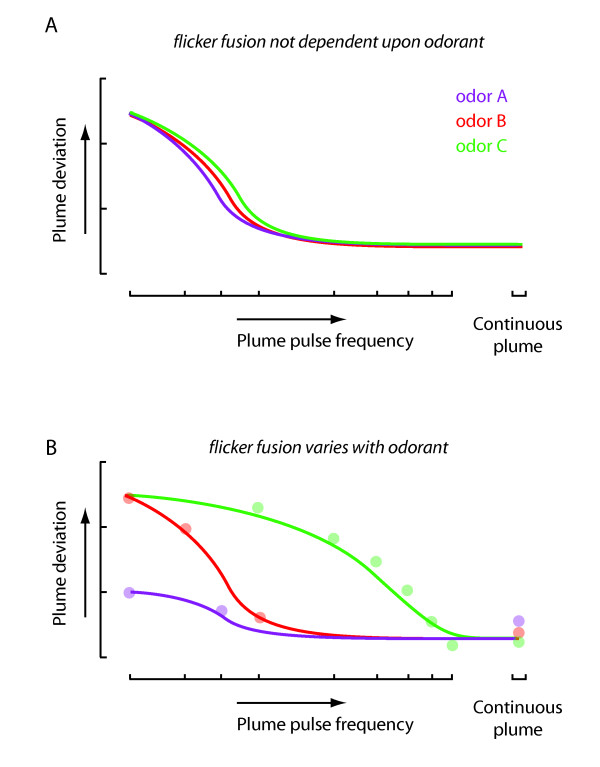
**Olfactory behavioral flicker-fusion is dependent on odor type**. (A) Hypothetical psychometric graph of plume deviation as a function of odor pulse frequency if different ORN temporal properties are 'fused' leading to the same behavioral responses regardless of odor type. Data are plotted in transparent shading. (B) Hypothetical psychometric graph of plume deviation as a function of odor pulse frequency if temporal properties propagate to downstream behavioral processes giving rise to flicker-fusion thresholds that differ with odor type.

Our results show that behavioral fusion of a flickering odor stimulus varies with odorant in a manner similar to wavelength dependence for human visual flicker fusion. The mechanism that explains the odorant dependent fusion of an intermittent stimulus may well reflect the temporal dynamics of higher-order neuronal processing that allow for efficient use of olfactory coding space [7,24,25]. We have shown that peripheral processing of temporal odor cues by antennal sensory mechanisms alone is not sufficient to explain the behavioral temporal fusion rates that we observe. If the temporal properties of an odor were effectively filtered or if flies could effectively track the temporal variations of any attractive odorant equally well, then we would expect similar behavioral performance across frequency regardless of odor type (Figure 8A). Furthermore, if the temporal frequency sensitivity of ORNs were placing the fundamental limit on the integration of a fluctuating odor signal, then we would expect that the tracking behavior would match the frequency sensitivity of the antennal field potential recordings, which represents a summation of the repertoire of ORN activity in the antenna[23]. However, we show that antennal sensory signals encode a temporal sequence of odor ON and OFF transients at a rate significantly higher that the rather low pulse frequencies at which tracking behavior diminishes. This suggests that the effectiveness with which a fly can track temporally intermittent odor signals is not constrained by the early sensory pathway (Figure 8B), but rather by higher-order processing stages. This is in contrast with pheromone plume tracking in moths where upwind flight can cease before the plume source is reached [26]. One plausible explanation for this change in tracking behavior is the reduction in peripheral sensory signaling (leading to fusion) when the filament encounter rate exceeds the temporal resolution limit of the input circuitry [26]. In addition, it has been shown that the cessation of receptor activity causes a change in flight pattern from upwind tracking to crosswind casting in moths [27].

Since the navigating fly is likely to encounter odor blends rather than a single scent [2], a low-pass temporal filtering mechanism could act to 'smooth out' the unpredictable chaotic distribution of odor stimuli, and thus confer an adaptive edge to a navigating insect to fly straighter through a circuitous plume.

### Persistent ethyl butyrate response cannot be attributed to the odor delivery system, and engages known visual dependence

We were surprised by the finding that flies continue to orient toward the odor nozzle for many seconds after the ethyl butyrate plume is terminated (Figure 5). Several lines of evidence suggest that the representation of ethyl butyrate must persist in central rather than peripheral circuits. First, electrophysiological responses of olfactory receptor neurons sensitive to ethyl butyrate faithfully track rapid stimuli (Figure 6C). Thus, persistent tracking behavior cannot be explained by either residual flow from the olfactometer or receptor saturation. In many insects, neurons upstream of the primary receptors show long-lasting responses to olfactory stimuli. For example, similar long-lasting excitation effects have been observed in recordings from protocerebrum olfactory neurons in moths (*Manduca sexta*) where the response was found to persists 30 seconds beyond the stimulus offset [28], and in the same species where the duration of an excitatory burst from PNs to a pulse of pheromone exceeded the duration of the stimulus [29]. Extracellular recordings made from the honey-bee brain have showed a rebound effect which lasts between 0.5 to several seconds after stimulus offset [30]. This persistent neuronal response long after cessation of the stimulus has been proposed to be the mechanistic reason behind casting flight behavior - a long term pheromone mediated response which occurs after 400-500 ms after loss of odor in several moth species that persists when the moth moves into clean air [31]. It is tempting to speculate that a similar mechanism could explain the persistency of the ethyl butyrate response after odor loss.

In addition, we have shown that during the tracking of the terminated ethyl butyrate plume, flies actively integrate visual cues, as their ability to track the plume is significantly diminished upon switching on a featureless uniform visual landscape (Figure 7A). These results are consistent with previous findings showing that a fly positioned within an active continuous plume requires rich visual feedback to maintain position at the plume [18]. This results in part from odor enhanced sensitivity to wide-field yaw rotation [32]. Here, we observe that the tracking persistence to the terminated ethyl butyrate plume is lost upon removal of yaw optic flow, suggesting that the same cross-modal mechanisms that are engaged to enable plume tracking persist beyond its termination. The fly continues to perceive and track a plume well after it has been terminated.

### Evolutionary perspectives

What would be the selective advantage of low versus high temporal fusion rates? A low fusion rate, such as is found for ethyl butyrate, could be advantageous when the distribution of odor signals is sparse and encountered infrequently, as would be the case far downwind from the source. In such a scenario, odor evoked changes in saccade rate and amplitude, coupled with upwind increase in flight velocity, would persist beyond the termination of the signal and combine to increase straight upwind flight and enhance the likelihood of encountering another odor packet in turbulent wind [27]. The cost to sluggish temporal fusion is that signal loss is not detected for some time. Sensitivity to higher frequency intermittency, such as is found with apple cider vinegar, could be advantageous upon approaching the source of an odor where signals are densely distributed, and thus higher sensitivity to the rapid loss of a plume would rapidly modify saccade dynamics and reduce the likelihood of overshooting the source.

Ethyl butyrate has been shown to be released by ripe fruits that could offer potent cues for ovipositional preference [33]. Thus, one could posit that flies might benefit from having a low flicker-fusion threshold (or a prolonged response after odor- loss) to ethyl butyrate thereby persevering within a sparse ethyl butyrate plume in order to maintain steady upwind heading and facilitate to reacquire a lost odor filament. It would be interesting to see if there are any differences in ovipositional preferences among the three odors tested. Positional repulsion to vinegar is mediated by the fly olfactory system [19,34] whereas ovipositional attraction to acetic acid is mediated by the fly's gustatory system [34]. Joesph et al, also postulate that fruit flies have an innate olfactory positional repulsion to acetic acid and that this repulsion is reversed only when flies need to oviposit [34]. Thus, we reason that a high fusion rate for vinegar could inform a navigating fly about the proximity of a food resource (a rotting fruit for example), where the position aversional olfactory circuits have been overridden by the gustatory system in order for the fly to deposit eggs.

However, these evolutionary perspectives are entirely speculative. Our results conclusively show that for a temporally fluctuating signal, some odors are more easily tracked at a distance and others potentially better tracked near the source. Since the dynamical properties of fluid flow entirely determine the spatial distribution of components of a blend, odor signals are reduced in intensity as distance from the source increases, but the relative ratios of molecules within the blend do not vary [4]. There is no physical mechanism by which some volatiles might be carried further from the source and therefore, there is no obvious way to explain a selective advantage of having differing behavioral sensitivity to the intermittency of different odors. Perhaps what we observe behaviorally bestows no clear evolutionary advantage, but rather results from the dynamical properties of olfactory coding that expand the perceptual odor space [7,25], but at the same time place important constraints on the ability of flies to track natural plumes.

## Conclusions

Our data provide new insights into the temporal processing of turbulent olfactory plumes of different fruit odors. Using a behavioral 'virtual-reality' assay, we have shown that the navigational ability of a fly to track a temporally intermittent plume is dependent on odor type, roughly analogous to human visual flicker fusion threshold which is dependent on the wavelength of the stimulus. Interestingly, flies display no innate preference between the specific odors tested under continuous odor conditions. We have defined a unique critical flicker fusion threshold value for each odor beyond which tracking behavior is no different than the tracking of a continuous plume of odor. Our study provides a framework for understanding the levels of processing of temporal information, as we show that downstream behavioral responses to intermittent odor stimuli are sluggish compared to peripheral processing as measured by antennal field potential responses.

## Methods

### Magnetic tether assay and odor delivery

Detailed procedures for constructing the components of the magnetic tether assay, odor delivery system, rearing of flies and acquiring video are described elsewhere in detail[18,35,36]. Briefly, adult female flies less than one week old were starved for 4-6 hours before being glued to a minutin pin (Fine Science Tools) and suspended between two magnets in such a way that allowed free rotation of the fly in the yaw plane. An electronic visual display panel system [37] consisting of LEDs enveloped the fly in azimuth, and occupied ±60 degrees in visual elevation with respect to the visual horizon. A gas multiplexer (Sable Systems) was used to switch mass flow regulated air precisely between two solenoid valves delivering odor or water vapor respectively at the rate of 7 ml per min. The frequency of odor pulses mentioned in the text and figures was controlled by custom code written in Matlab. The odor port was positioned less than 3 mm away from the head of the fly. A suction chamber (13 l/min) at the base of the arena ensured that the odor plume was drawn away quickly. Body heading of the fly in flight was illuminated with infrared and digitized using an infrared firewire camera (Fire-i) and analyzed offline using custom Matlab scripts. Odor stimuli consisted of apple cider vinegar (grocery store brand from Ralphs), natural ethanol extract of banana (Polarome Inc), and ethyl butyrate (99% pure, Sigma-Aldrich).

Each experimental trial began with the fly oriented within the plume. This was accomplished by projecting a 30^0 ^vertical bar on the LED display near the odor nozzle and oscillating the bar at 2 Hz for 10 seconds. Flies show a strong bar fixation reflex, and quickly respond to the bar motion by orienting directly toward the bar, at the odor nozzle (Figure 1a)

### Photoionization Detector (PID) measurements

To measure the temporal properties of odor stimuli, we used a miniature photoionization detector (mini-PID Aurora Scientific, Ontario, Canada). The tip of the device inlet probe was placed at the location of the fly's head during flight, 2 mm from the odor nozzle. Ethanol, which has an ionization-potential of 10.62 eV, was used as a tracer gas.

### Electrophysiology

Antennal Field Potential (AFP) recordings or electroantenograms (EAGs) were made from the third antennal segment of *D.melanogaster *as described previously [23]. Briefly, a female fly was trapped in a truncated 200 μl micropipette tip in such a way that the olfactory organs were exposed. Recording and ground electrodes were filled with 0.17 M NaCl and the ground electrode was inserted into the head capsule. The recording electrode was brought into electrical contact with the dorso-medial surface of the funiculus and the odor delivery system consisted of solenoid valves controlled by custom written Matlab script that delivered ethyl butyrate pulses at the rate of 2.5 Hz alternating with water vapor. Signals were acquired at 10 kHz and filtered at 2 kHz using A-M systems amplifier (Model 3600).

## Authors' contributions

PK, BD and MF conceived and designed the experiments. PK and BD performed the experiments. PK and BD analyzed the data. PK and MF wrote the paper. All authors read and approved the final manuscript.

## Supplementary Material

Additional file 1 **Method to determine the range of odor pulse frequencies for testing individually**. To determine the window of odor frequencies that the critical flicker fusion threshold value might lie within we subjected the same set of flies to receive either a frequency modulated plume where each odor was presented from frequencies ranging from 10 Hz to 0.1 Hz or a continuous plume of the same odorant. For apple cider vinegar, the trajectory of plume deviation values for flies that received the sweep-pulsed vinegar plume diverged at roughly 4.6 seconds from the trajectory of mean plume deviation in response to the continuous plume. For banana, the point of deviation between the two stimulus conditions was about 11.6 seconds. For ethyl butyrate, a monomolecular odorant, the point of deviation occurred at 12.4 seconds. Odor frequencies that were bracketed within this range were then presented individually as shown in Figure 4. Click here for file
