# Supplementary material for: Odor identity influences tracking of temporally patterned plumes in Drosophila
Source: BMC Neurosci. 2011 Jun 27;12:62. doi: 10.1186/1471-2202-12-62 (PMC3145592; doi:10.1186/1471-2202-12-62)
Supplement: Additional file 1 — Method to determine the range of odor pulse frequencies for testing individually. To determine the window of odor frequencies that the critical flicker fusion threshold value might lie within we subjected the same set of flies to receive either a frequency modulated plume where each odor was presented from frequencies ranging from 10 Hz to 0.1 Hz or a continuous plume of the same odorant. For apple cider vinegar, the trajectory of plume deviation values for flies that received the sweep-pulsed vinegar plume diverged at roughly 4.6 seconds from the trajectory of mean plume deviation in response to the continuous plume. For banana, the point of deviation between the two stimulus conditions was about 11.6 seconds. For ethyl butyrate, a monomolecular odorant, the point of deviation occurred at 12.4 seconds. Odor frequencies that were bracketed within this range were then presented individually as shown in Figure 4. [file 1471-2202-12-62-S1.DOCX]

**Supplementary Methods**

To determine the window of odor frequencies that the critical flicker fusion threshold value might lie within we subjected the same set of flies to receive either a frequency modulated plume where each odor was presented from frequencies ranging from 10Hz to 0.1Hz or a continuous plume of the same odorant. For apple cider vinegar, the trajectory of plume deviation values for flies that received the sweep-pulsed vinegar plume diverged at roughly 4.6 seconds from the trajectory of mean plume deviation in response to the continuous plume. For banana, the point of deviation between the two stimulus conditions was about 11.6 seconds. For ethyl butyrate, a monomolecular odorant, the point of deviation occurred at 12.4 seconds. Odor frequencies that were bracketed within this range were then presented individually as shown in Figure 4.
